# Adductome-based identification of lysine monomethylation as a key post-translational protein modification in autoimmune diseases

**DOI:** 10.1016/j.jbc.2025.110684

**Published:** 2025-09-04

**Authors:** Kosuke Yamaguchi, You-Yun Hu, Kaito Kawajiri, Masanori Itakura, Fumie Nakashima, Takahiro Shibata, Koji Uchida

**Affiliations:** 1 Graduate School of Agricultural and Life Sciences, The University of Tokyo, Tokyo, Japan; 2 Graduate School of Bioagricultural Sciences, Nagoya University, Nagoya, Japan

**Keywords:** autoimmune diseases, systemic lupus erythematosus, post-translational modification, adductome, lysine monomethylation, histone, B-cell differentiation

## Abstract

Post-translational modifications (PTMs) of proteins are efficient biological mechanisms for expanding the genetic code and for regulating cellular physiology. However, there have been no systematic approaches to profile all the PTMs critical for autoreactive neoantigen production or the etiology and pathology of autoimmune diseases. In the present study, to gain insight into protein PTMs associated with systemic lupus erythematosus (SLE), we applied a mass spectrometry–based method for the comprehensive analysis of modified amino acids (“adductome”). By comprehensively measuring modified lysines and histidines in mouse tissue homogenates from the control and SLE-prone MRL-lpr mice, we observed a significant decrease in lysine monomethylation as a unique alteration in the PTMs of SLE mice splenocytes. One of the targets for the downregulation of lysine monomethylation was identified as histone H3. Downregulation of histone H3 lysine 4 monomethylation (H3K4me1) was also observed in the B cells from SLE mice. Inhibition of the H3K4me1 demethylase LSD1 suppressed the differentiation of naive B cells into antibody-secreting cells. In addition, the reduction of H3K4me1 in SLE mice resulted in the downregulation of PAX5, a transcription factor indispensable for the maturation of B cells, leading to the activation of antibody-secreting cells. The results of this study suggest that dysregulation of histone lysine monomethylation may play a significant role in the pathophysiology of SLE and therefore may be a target for epigenetic-based lupus treatment.

Most proteins in the human body undergo a wide range of reversible post-translational modifications (PTMs), such as phosphorylation, methylation, and glycosylation. They are enzyme-catalyzed and constitutively important to fulfill specific structural or functional roles or to allow efficient recycling of amino acid constituents and are efficient biological mechanisms for expanding the genetic code and for regulating cellular physiology. PTMs are introduced at a variety of amino acid residues, including the nucleophilic side chain of lysine, one of the three basic residues important for protein structure and function. The PTMs of lysine residues have been proven to be key regulators of gene expression, protein–protein interactions, and protein processing and degradation. Particularly, the discovery of acetylation and methylation of histone proteins dramatically changed our understanding of gene regulation and beyond. On the other hand, PTMs are a critical mechanism that alters protein structure to generate neoantigens and induce subsequent autoimmune responses. In rheumatoid arthritis patients, for example, it has been previously demonstrated that citrullinated peptides are a critical source of neoantigens (1). Recent studies have also shown that the carboxyethylation of cysteine residues in integrin αIIb induces autoantibody production in ankylosing spondylitis (2). The phosphorylated glial cell adhesion molecule showed a strong affinity for autoantibodies established from multiple sclerosis patients (3). Thus, dysregulated PTMs may trigger various autoimmune diseases.

Systemic lupus erythematosus (SLE) is a potentially fatal systemic autoimmune disease characterized by the excessive production of autoantibodies. Of the multiple autoantibodies reported in SLE, those recognizing the native DNA are the best known (4). The appearance of anti-DNA antibodies in humans and murine models of lupus correlates with the progression of the disease (5, 6). Specifically, compared with all other lupus autoantibodies, antibodies against DNA are thought to be the most pathogenic and involved in the development of renal pathology (5). Although nuclear materials, such as DNA and nucleosomes, released from apoptotic cells are considered to be the main pathway for anti-DNA antibody production, other pathways, such as necrosis, necroptosis, pyroptosis, and neutrophil extracellular traps, may also be potential sources of extracellular DNA in patients with SLE (7). However, because of the systemic character and complexity of the disease, it remains unclear what exactly are the primary stimuli that drive such autoantibody responses and the mechanisms that regulate the entire pathological process in SLE.

There have been few systematic approaches to profile all the PTMs critical for autoreactive neoantigen production or the etiology and pathology of autoimmune diseases. In the present study, to gain insights into protein PTMs associated with SLE, we adapted an adductome analysis, which is a mass spectrometry (MS)–based method for the comprehensive analysis of covalently modified PTMs (adducts). This technique is based on the principle that the fragmentations of covalently modified peptides or amino acids produce the precursor-specific immonium ions. Previous studies have suggested strategies to detect peptides containing specific adducts, such as phosphorylation, methylation, acetylation, formylation, ubiquitylation, and SUMOylation, by scanning adduct-derived immonium ions using triple-quadrupole MS (8, 9, 10). On the other hand, we focused on authentic amino acid–derived immonium ions other than specific PTM-derived ones. Based on the fact that the lysine- and histidine-derived adducts commonly generate authentic amino acid–derived immonium ions, we developed an MS-based comprehensive analysis method to detect lysine- or histidine-derived adducts (11). In this method, samples are hydrolyzed with acid or enzymes to free amino acids, which are then subjected to MS. Spectral analysis using selected reaction monitoring (SRM) allows profiling of lysine- or histidine-derived adducts. Although adductome analysis cannot identify the exact structure of PTMs because it only has unit resolution, it is a powerful tool for comprehensively searching PTM candidates.

In this study, this adductome approach enabled a systematic screening of SLE-associated PTMs and confirmed reduced lysine monomethylation in the spleen of SLE-prone mice. In addition, we identified histone H3 as the target of the lysine monomethylation. Downregulation of histone H3 monomethylation was found to occur in B cells, and monomethylation at histone H3 lysine 4 (H3K4me1) was reduced in B cells from SLE mice. Inhibition of H3K4 demethylase reduced the B-cell differentiation into antibody-secreting cells (ASCs). Our discovery of the reduction of H3K4me1 associated with SLE suggests that epigenetic interventions might be one of the effective therapeutic methods against SLE and other autoimmune diseases.

## Results

### Comparative and comprehensive analysis of PTMs in tissue homogenates from wild-type and SLE mice

**Figure 1:**
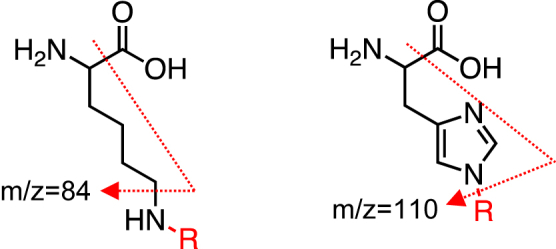
**Fragmentation of lysine and histidine adducts**.

**Figure 2:**
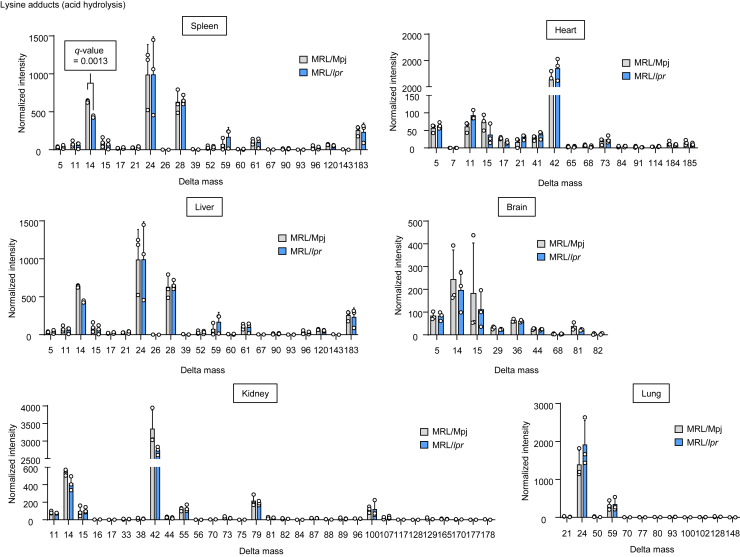
**Adductome analysis of lysine adducts from acid-hydrolyzed samples.** The delta mass clusters in each of the tissue samples (n = 3). Among the delta mass shifts of −21 to 203, only those that could be detected are shown. Statistical significance between each group was determined using a multiple *t* test.

**Figure 3:**
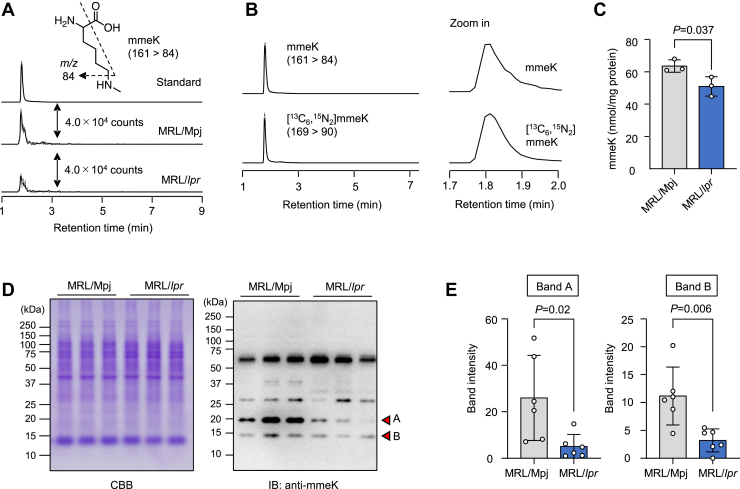
**The reduction of mmeK-containing proteins in the spleen of MRL-lpr mice.***A*, LC–ESI-MS/MS analysis of mmeK in the mouse spleen. Representative LC–ESI–MS/MS chromatograms of standard (*upper trace*), MRL-Mpj mouse spleen–derived mmeK (*middle trace*), and MRL-lpr mouse spleen–derived mmeK (*lower trace*) are shown. *B*, LC–ESI–MS/MS analysis of mmeK and isotope-labeled mmeK. *Left panel,* representative LC–ESI–MS/MS chromatograms of mmeK (*upper trace*) and isotope-labeled mmeK (*lower trace*) are shown; *right panel*, zoomed-in chromatograms of *left panel* are shown. *C*, quantitative identification of mmeK in the spleen of MRL-Mpj and MRL-lpr mice. The mmeK was quantified using LC–ESI–MS/MS coupled with a stable isotope dilution method. The data are shown as the mean ± SD (n = 3, biologically independent experiments). Student’s *t* test (two-sided). *D*, immunoblotting showing mmeK-containing protein levels in the spleen of MRL-Mpj and MRL-lpr mice. *Left panel,* SDS-PAGE and Coomassie Brilliant Blue (CBB) staining; *right panel,* immunoblot analysis with anti-mmeK antibody. *E*, quantification of band A (*left panel*) and band B (*right panel*) shown in *D*. The data are shown as the mean ± SD (n = 3, biologically independent experiments). Student’s *t* test (two-sided). ESI, electrospray ionization; mmeK, *N*^ε^-monomethyl-l-lysine.

Taking advantage of the fact that the authentic lysine and histidine commonly generate specific fragment ions at *m/z* 84 and 110 (Fig. 1), we comprehensively analyzed their modified forms in mouse tissue homogenates from the control and SLE-prone mice. We hydrolyzed each tissue’s proteins under acidic conditions and subjected them to adductome analysis (Fig. S1). As shown in Figures 2 and S2, we detected several delta mass shifts, probably because of PTMs, between the control and SLE mice. Although we could not rule out the possibility that some of these mass shifts were noise, we observed a statistically significant reduction in the mass shift of Lys +14 in the spleen of MRL-lpr mice. As acid hydrolysis may result in cleavage of amide linkage–containing PTMs, we also hydrolyzed each tissue’s proteins by enzymatic digestion. Several mass shifts were matched with dipeptides; for example, His +57 is likely to be dipeptides formed from histidine and glycine, and His +147 is likely to be dipeptides formed from histidine and oxidized methionine (Fig. S3). These mass shifts indicated that the enzymatic digestion might be incomplete, but there were no differences between the control and SLE mice. Although there were no statistically significant differences in enzymatic digestion experiments, the mass shifts of Lys +14 and Lys +42 were indicated to be reduced in the spleen of MRL-lpr mice (Fig. S4). The mass shifts of Lys +14 and Lys +42 are supposed to be due to lysine monomethylation and acetylation, respectively. Decreased lysine acetylation in autoimmune diseases is consistent with the previous findings that (i) the levels of histone H3 and H4 lysine acetylation were reduced in MRL-lpr mice compared with the control mice and (ii) the inhibition of histone deacetylase mitigated the B-cell-driven autoimmune conditions of SLE (12, 13). Because of these extensive studies about lysine acetylation in SLE, we decided to focus on the mass shift of Lys +14, which was observed to be downregulated in the SLE mice (Fig. 3*A*). Based on the LC–electrospray ionization (ESI)–MS/MS analysis of the authentic stable isotope–labeled sample, the product with the molecular ion at *m/z* 161 was identical to *N*^ε^-monomethyl-L-lysine (mmeK). Using isotope dilution–based LC–ESI–MS/MS, we quantitatively analyzed mmeK and confirmed that lysine monomethylation was decreased in the SLE mice compared with the controls (Fig. 3, *B* and *C*).

### Identification of mmeK-containing proteins

We next sought to identify the mmeK-containing proteins that were observed to be decreased in the spleen from the SLE mice. The immunoblot analysis using mmeK-specific antibodies detected four protein bands in the SLE mice, of which two bands (A and B) were significantly decreased (Fig. 3, *D* and *E*). The result and the observation that the decrease in these two bands in the SLE mice was observed in both the 20- and 39-week-old mice (Fig. S5) suggested that the reduction of lysine monomethylation in band A and B proteins may be associated with the pathogenesis of SLE. Given the significantly decreased levels of the mmeK-containing proteins in the spleen of the MRL-lpr mice, we next sought to identify the mmeK-containing protein that was observed to be decreased in the SLE mice. To this end, splenic lysates were separated and fractionated by size-exclusion chromatography, and the levels of mmeK in each fraction were assessed by a method using isotope dilution–based LC–ESI–MS/MS (Fig. S6*A*). Compared with the control mice, although the changes were not statistically significant, mmeK levels in the SLE mice were lower in fractions 10 and 13 (Fig. S6*B*). In addition, we confirmed that fraction 13 contained an mmeK-containing protein corresponding to band B (Figs. S6*C*, and S7, *A* and *B*). The proteomic analysis of fraction 13 identified hemoglobin as one of the candidate band B proteins (Table S1). Since hemoglobin is a major protein in red blood cells (RBCs), we analyzed the lysine monomethylation of hemoglobin in RBCs from the control MRL-Mpj mice and found that circulating RBCs in their blood also contained mmeK (Fig. S7, *C* and *D*). In addition, the lysine monomethylation of hemoglobin was decreased in the RBCs from MRL-lpr mice, which was consistent with the results from the spleen lysates. These data suggest that the band B protein is hemoglobin. However, identification of the monomethylation sites in hemoglobin was unsuccessful in both the spleen and RBC samples.

**Figure 4:**
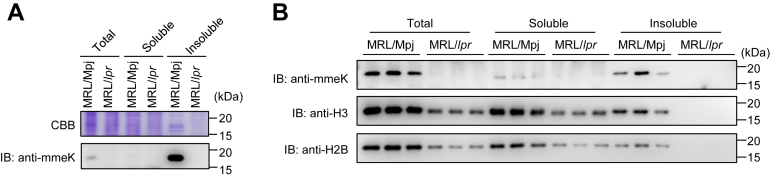
**Identification of mmeK-containing proteins.***A*, separation of mmeK-containing proteins (band A) by centrifugation. The splenic lysates were centrifuged by 12,000*g* for 5 min and separated into soluble and insoluble fractions. *Upper panel,* SDS-PAGE and Coomassie Brilliant Blue (CBB) staining; *lower panel,* immunoblot analysis with anti-mmeK antibody. *B*, immunoblotting showing mmeK-containing histones in the insoluble fraction. *Upper panel,* immunoblot analysis with anti-mmeK antibody; *middle panel,* immunoblot analysis with anti-histone H3; and *lower panel,* immunoblot analysis with anti-histone H2B. mmeK, *N*^ε^-monomethyl-l-lysine.

On the other hand, we were unable to detect any mmeK-containing proteins derived from band A in the size-exclusion chromatography experiments (Fig. S6). We hypothesized that the centrifugation to remove aggregates prior to the size-exclusion chromatography might result in the deposition of the band A protein. Hence, spleen lysates from both the MRL-Mpj and MRL-lpr mice were separated into soluble and insoluble fractions and immunochemically analyzed, showing that the mmeK-containing protein corresponding to band A was present in the insoluble fraction from the control mice (Fig. 4*A*). In addition, proteomic analysis of band A identified molecular weight–matching several proteins, including histone H2B, histone H3, and myosin (Table S2). As lysine methylation of histone is crucial for shaping the epigenetic landscape and is also relevant to cell physiology, we focused on histones H2B and H3 for further analysis. Consistent with the presence of mmeK in the insoluble fraction from the control mice, both H2B and H3 were detected in the same insoluble fractions from the control mice but not from the SLE mice (Fig. 4*B*).

### Identification of lysine monomethylation sites in histone H3

**Figure 5:**
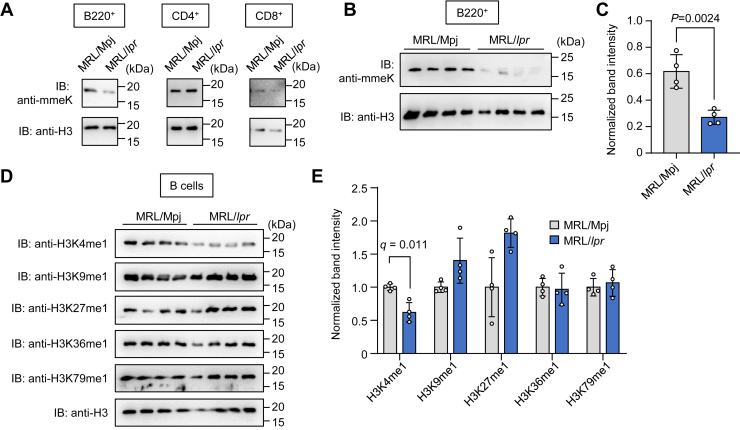
**H3K4me1 is decreased in B cells of MRL-lpr mice.***A*, immunoblotting showing mmeK-containing proteins in the splenic immune cells. *Left panel,* immunoblot analysis of B cells; *middle panel,* immunoblot analysis of CD4^+^ T cells; *right panel,* immunoblot analysis of CD8^+^ T cells. *B*, immunoblotting showing mmeK-containing proteins in B cells. *Upper panel,* immunoblot analysis with anti-mmeK antibody; *lower panel,* immunoblot analysis with anti-histone H3. *C*, quantification of mmeK-containing proteins shown in *B*. The mmeK-containing protein band intensities were measured and normalized to histone H3. The data are shown as the mean ± SD (n = 4, biologically independent experiments). Student’s *t* test (two-sided). *D*, immunoblotting showing mmeK-containing histone H3 in B cells. *E*, quantification of mmeK-containing histone H3 shown in *D*. mmeK-containing histone H3 band intensities were measured and normalized to total histone H3. The data are shown as the mean ± SD (n = 4, biologically independent experiments). Statistical significance between each group was determined using a multiple *t* test. H3K4me1, histone H3 lysine 4 monomethylation; mmeK*, N*^ε^-monomethyl-l-lysine.

Based on the identification of histones as mmeK-containing proteins, we first sought to identify the subset of splenocytes that exhibit downregulation of the histone lysine monomethylation in the SLE mice. Using magnetic-activated cell sorting (MACS) methods, the splenocytes were separated into three major subsets of immune cells, including B (B220^+^) cells, CD4^+^ T cells, and CD8^+^ T cells. After the separation, we subjected each subset to an immunoblot analysis against histone H3 because it is a well-known target for lysine monomethylation (14). The results clearly showed that lysine monomethylation of H3 was downregulated in B cells from the MRL-lpr mice (Fig. 5, *A*–*C*). We then attempted to identify the lysine monomethylation sites in H3. Of the five methylation sites in H3 (14), downregulation of H3K4me1 was observed in the B cells from the MRL-lpr mice (Fig. 5, *D* and *E*). Although there are three lysine methylation states (mono-, di-, and trimethylations), no changes were observed in the levels of the H3K4 dimethylations and trimethylations between the control and SLE mice (Fig. S8). Thus, downregulation of the histone H3 monomethylation was found to occur in the B cells.

### Involvement of H3K4 methylation/demethylation enzymes

The histone methylation levels are precisely regulated by the enzymatic action of specific methyltransferases and demethylases. To gain insight into the responsible enzymes in reducing the H3K4me1 levels in the SLE mice, we analyzed the mRNA expression levels of the H3K4 methyltransferases (*Mll1*, *Mll2*, *Mll3*, *Mll4*, *Smyd1*, *Smyd2*, *Set1a*, *Set1b*, *Set7/9*, and *Prdm9*) and demethylases (*Lsd1*, *Lsd2*, *Riox1*, *Jarid1a*, *Jarid1b*, *Jarid1c*, and *Jarid1d*). Among the detectable H3K4 methyltransferases, only the *Prdm9* transcripts were significantly decreased in the SLE mice compared with the controls, whereas several H3K4 methyltransferases (*Mll1*, *Mll4*, *Smyd2*, *Set1A*, and *Set7/9*) were upregulated in the SLE mice (Fig. S9*A*). On the other hand, among the detectable H3K4 demethylases, two H3K4 demethylase transcripts, *Lsd1* and *Riox1*, were significantly increased in the MRL-lpr mice, whereas no significant changes in other demethylase genes were observed (Fig. S9*B*). Therefore, it is likely that the overall balance between the methyltransferases and demethylases leads to decreased histone methylation; however, the detailed correlations remain unclear.

**Figure 6:**
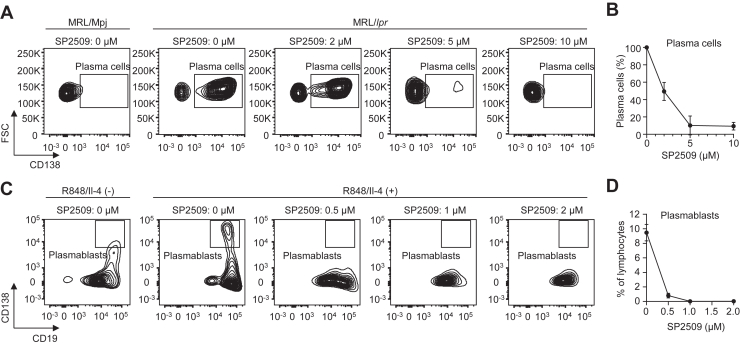
**Inhibition of H3K4 demethylase LSD1 suppresses the differentiation of B cells.***A*, representative flow cytometry plots of plasma cells. Splenocytes from MRL-Mpj mice or MRL-lpr mice were cultured with SP2509 (indicated concentration) for 48 h. The populations of the plasma cells were measured by flow cytometry. *B*, quantification of plasma cells shown in *A*. The populations of the plasma cells without SP2509 were regarded as 100%. The data are shown as the mean ± SD (n = 3, biologically independent experiments). *C*, representative flow cytometry plots of plasmablasts. The B cells from BALB/c mice were stimulated with R848/IL-4 and cultured with SP2509 (indicated concentration) for 48 h. The populations of the plasmablasts were measured by flow cytometry. *D*, quantification of plasmablasts shown in *C*. The populations of the plasmablasts are shown as the percentage of lymphocytes. The data are shown as the mean ± SD (n = 3, biologically independent experiments). H3K4, histone H3 lysine 4.

To gain more insight into the involvement of the H3K4 methylation–demethylation enzymes, we tested the effect of the H3K4 demethylase inhibitor on the differentiation of the B cells into ASCs. When splenocytes isolated from MRL-lpr mice were treated with SP2509, an inhibitor of H3K4 demethylase LSD1, a dose-dependent decrease in the population of plasma cells was observed (Fig. 6, *A* and *B*). Concomitantly, the levels of immunoglobulin G (IgG) and immunoglobulin M (IgM) secreted in the culture medium were also reduced by the inhibitor (Fig. S10, *A* and *B*). In addition, the effect of the H3K4 demethylase inhibitor on differentiation of the BALB/c mouse B cells induced by R848, an agonist of Toll-like receptor 7 and 8, and interleukin 4 (IL-4) (R848/IL-4) was also tested. Treatment with R848/IL-4 promoted a remarkable differentiation of the B cells into plasmablasts, whereas the H3K4 demethylase inhibitor dose-dependently reduced the proportion of plasmablasts (Fig. 6, *C* and *D*). The inhibitor also suppressed the secretion of IgM in the culture medium (Fig. S10*C*). These data suggest that altered expression levels of an H3K4 demethylase may be, at least in part, responsible for the reduction of H3K4me1 in the SLE mice.

### Molecular basis linking H3K4me1 and B-cell maturation

**Figure 7:**
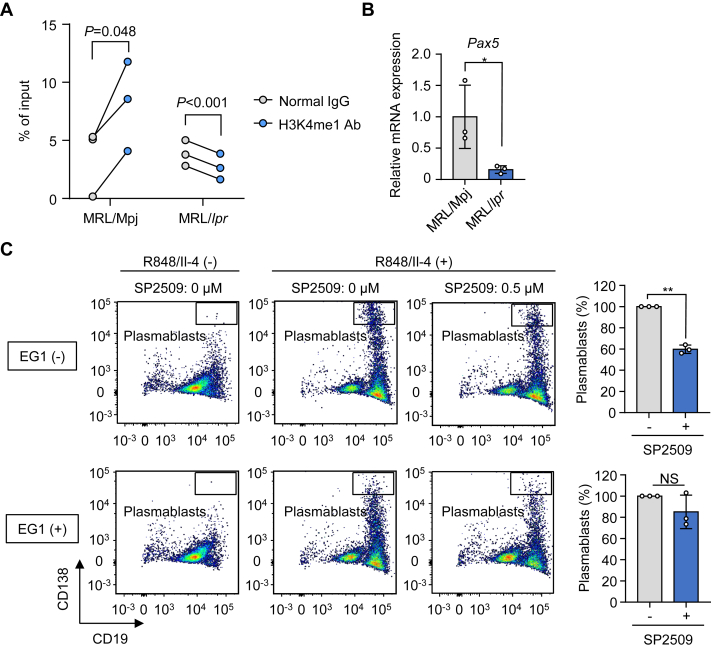
**H3K4me1–PAX5 axis regulates the differentiation of B cells.***A*, ChIP–qPCR analyses showing the interaction of H3K4me1 and PAX5 enhancer region (n = 3, biologically independent experiments). Paired *t* test (two-sided). *B*, quantification of the *Pax5* transcripts. The mRNA expression levels of *Pax5* were measured and normalized to *Actb*. The data are shown as the mean ± SD (n = 3, biologically independent experiments). Student’s *t* test (two-sided). ∗*p* < 0.05. *C*, PAX inhibitor EG1 cancels the differentiation-suppressing effects of SP2509. The B cells from BALB/c mice were stimulated with R848/IL-4 and cultured with SP2509 (indicated concentration) and EG1 for 48 h. *Left panel,* representative flow cytometry plots of the plasmablasts; *right panel,* quantification of the plasmablast populations. The populations of the plasmablasts without SP2509 were regarded as 100%. The data are shown as the mean ± SD (n = 3, biologically independent experiments). Student’s *t* test (two-sided). ∗∗*p* < 0.01. ChIP, chromatin immunoprecipitation; H3K4me1, histone H3 lysine 4 monomethylation; IL-4, interleukin 4; qPCR, quantitative PCR.

H3K4me1 is regarded as a canonical mark of both the poised and active transcriptional enhancers. Particularly, with regard to the B cells, H3K4me1 is associated with enhancer regions of the *Pax5* gene (15). PAX5 is a transcription factor indispensable for the maturation of B cells, and the loss of PAX5 promotes plasma cell differentiation (16). Therefore, we speculated that the loss of H3K4me1 reduces the PAX5 expression and promotes the overdifferentiation of B cells into ASCs. To prove this hypothesis, we conducted chromatin immunoprecipitation (ChIP) and successively isolated H3K4me1-containing chromatin (Fig. S11). We then amplified the H3K4me1-interacting genomic DNA regions and confirmed that H3K4me1 is associated with the *Pax5* enhancer region in B cells isolated from the MRL-Mpj splenocytes (Fig. 7*A*). However, no interaction of H3K4me1 and *Pax5* was detected in B cells isolated from the MRL-lpr splenocytes. Consistent with the results of the ChIP–quantitative PCR (qPCR) assay, the expression levels of *Pax5* mRNA were downregulated in B cells isolated from the MRL-lpr splenocytes (Fig. 7*B*). To demonstrate that PAX5 is under the control of the H3K4 monomethylation, we treated B cells with EG1, a PAX inhibitor that inhibits the interaction of PAX with DNA and suppresses the expression of the PAX-targeted genes (17). As expected, the PAX inhibition with EG1 abolished the inhibitory effect of SP2509 on B-cell differentiation (Fig. 7*C*). Although the secreted IgM levels were suppressed by SP2509 in both the control and EG1-treated B cells, the IgM levels were maintained in the EG1-treated group (approximately 70%) compared with the control group (approximately 60%) (Fig. S12). These results indicated that PAX5 exists downstream of the H3K4me1-mediated B-cell maturation pathway and partially contributes to B-cell differentiation into ASCs. Taken together, these data suggest that the reduction of H3K4me1 may promote B-cell differentiation into ASCs *via* the downregulation of PAX5.

## Discussion

In the present study, to gain insight into protein PTMs associated with autoimmune diseases, we applied a strategy for the detection of modified amino acids associated with SLE. To identify the modified amino acids of proteins in the tissues from the wild-type and SLE mice, we measured the mass differences between the original amino acids (Lys and His) and the actual adducts (Fig. 1). Analysis of the modified lysine revealed a decrease in two products with molecular ions at *m/z* 189 and 161 ([M + H]^+^) in tissues from the SLE mice compared with the wild-type mice, corresponding to 42-Da and 14-Da increases in the mass value of lysine, respectively (Figs. 2 and S4). These mass shifts were supposed to be due to lysine acetylation and monomethylation, respectively. To the best of our knowledge, lysine acetylation in SLE has already been well studied, whereas few studies have been conducted regarding lysine methylation associated with SLE. Therefore, we focused on lysine monomethylation in the present study. Isotope dilution–based LC–ESI–MS/MS analysis of mmeK indeed confirmed that lysine monomethylation was significantly reduced in the SLE mice compared with the controls (Fig. 3, *B* and *C*).

Protein lysine methylation is one of the PTMs that influences many cellular pathways, including epigenetic regulations. The chemical reaction of lysine methylation is the reversible addition of one, two, or three methyl groups to the ε-nitrogen of a lysine side chain, resulting in the formation of monomethylated, dimethylated, and trimethylated derivatives, respectively. The addition of methyl groups to lysine residues is catalyzed by substrate-specific lysine methyltransferases (*writers*) with SAM, a major methyl group donor *in vivo*. Conversely, the removal of a methyl group is catalyzed by substrate-specific lysine demethylases (*erasers*) through an oxidative demethylation reaction. The extent of protein lysine methylation is regulated by the balanced activity of lysine methyltransferases and demethylases. Lysine methylation induces subtle changes in the size and electrostatic status of a lysine residue, but methylated lysine can be distinguished by downstream effector proteins (*readers*) that contain a methylated lysine reader motif. Reader proteins recognize specific methyl-lysine residues in addition to the adjacent amino acid sequence and the methylation state. Based on the findings that lysine monomethylation was significantly reduced in SLE mice, we attempted to identify the mmeK-containing proteins (bands A and B) that were observed to be decreased in the spleen from the SLE mice (Fig. 3, *D* and *E*). Size-exclusion chromatography followed by isotope dilution–based LC–ESI–MS/MS detected only one mmeK-containing protein corresponding to band B, which was identified as hemoglobin (Figs. S6, S7, and Table S1). Following the identification of hemoglobin, we observed the presence of mmeK in circulating RBCs from the control mice and reduced lysine monomethylation of hemoglobin in RBCs from the SLE mice (Fig. S7, *C* and *D*). Regarding lysine methylation of hemoglobin, there is a previous study showing that the methyl group from SAM is nonenzymatically transferred into hemoglobin (18). Therefore, the amounts of SAM may be a rate-limiting factor for hemoglobin methylation. In general, methylation of biomolecules, including protein and DNA, can be achieved by a specific methyltransferase and SAM. Therefore, the amounts of SAM could be one of the rate-limiting factors for methylation *in vivo*. However, previous studies reporting the amount of SAM in SLE patients are controversial. Oaks and Perl (19) have suggested that SLE patients had lower levels of GSH compared with healthy controls, leading to SAM depletion. They also indicated that the SAM deficiency might cause DNA hypomethylation in SLE patients (19). Moreover, the supplementation of SAM was shown to suppress the overproliferation of B cells, one of the etiologies of SLE (20). In contrast, Stojan *et al.* (21) reported that the level of SAM was higher in SLE patients than in healthy controls.

On the other hand, mmeK-containing proteins derived from band A were not detectable in size-exclusion chromatography experiments (Fig. S6), but they were detected in the insoluble fractions from the control mice but not from SLE mice and identified as histones H2B and H3 (Fig. 4 and Table S2). These histone proteins likely formed aggregates during preparation of the samples for size-exclusion chromatography and were present in the insoluble fraction. Histones are well-characterized substrates of lysine methylation, and the methylation mark of histone is tightly associated with epigenetic modulation of transcription. Dysregulation of protein lysine methylation is often associated with neurological disorders, developmental abnormalities, and cancer (22, 23, 24). The present study identified H3K4me1 as one of the SLE-associated PTMs in B cells. H3K4me1 is known as the mark of an enhancer, which supports the expression of the target genes. Primed enhancers are marked by H3K4me1 coupled with the depletion of histone H3 lysine 4 trimethylation (H3K4me3), whereas active enhancers are enriched for H3K4me1, H3K27 acetylation, H4K16 acetylation, and H3K122 acetylation. In addition, Kubo *et al.* (25) revealed that H3K4me1 facilitates the interaction of a promoter and enhancer to activate the target gene expression. Numerous studies have demonstrated that H3K4me1 is highly dynamic and correlates with the cell type–specific gene expression profiles. In the context of histone lysine methylation in SLE, significant changes in H3K4me3 have been demonstrated in several key candidate genes related to immune responses in peripheral blood mononuclear cells from SLE patients (26, 27).

Following the identification of H3K4me1 as an SLE-associated PTM in B cells, we sought to identify specific methylation/demethylation enzymes involved in the regulation of histone lysine methylation. As a result, we were able to narrow down a few candidate enzymes but were unable to identify them. Nevertheless, of the two H3K4 demethylase transcripts, *Lsd1* and *Riox1*, that were significantly upregulated in the SLE mice, we selected LSD1 because of the inhibitor availability and found that the H3K4 demethylase inhibitor (SP2509) reduced the number of ASCs derived from MRL-lpr splenocytes (Fig. 6, *A* and *B*). In addition, we also observed that differentiation of the BALB/c mouse B cells and the subsequent secretion of IgG and IgM in the culture medium were significantly suppressed by the inhibitor (Figs. 6, *C* and *D* and S10). LSD1 is essential for the proliferation and differentiation of naïve B cells into ASCs (28). In addition, plasmablasts lacking LSD1 display an increased H3K4me1 and chromatin accessibility at the naive B cells' active enhancers and the binding sites of transcription factors, such as Blimp-1, PU.1, and IRF4 (28). Thus, dysregulation of histone lysine methylation in SLE mice may be, at least in part, ascribed to the altered expression of LSD1.

**Figure 8:**
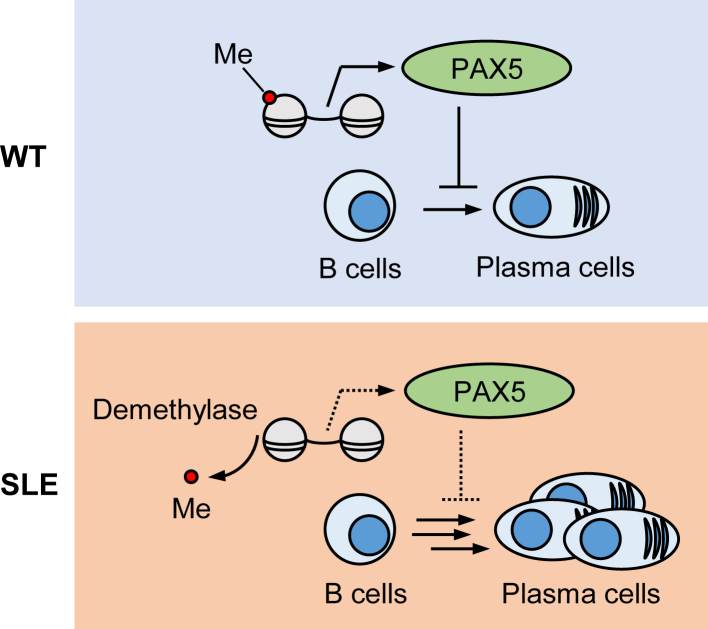
**A schematic model depicting the H3K4me1-involved SLE etiologies.** H3K4me1, histone H3 lysine 4 monomethylation; IL-4, interleukin 4; SLE, systemic lupus erythematosus.

H3K4me1 is associated with enhancer regions of the *Pax5* gene (29). PAX5 is involved in the inhibition of the ASC differentiation and antibody production (16). In this study, we observed that the interaction of the *Pax5* enhancer regions with the active transcription marker, H3K4me1, was abolished in the SLE mice (Fig. 7*A*). We therefore speculated that the dissociation of H3K4me1 and *Pax5* enhancer may result in the reduced *Pax5* mRNA expression in the SLE mice (Fig. 7*B*). In support of this hypothesis, PAX inhibition with EG1 suppressed the inhibitory effects of the LSD1 inhibitor, SP2509, on B-cell differentiation, suggesting that the PAX5 function is regulated by H3K4me1 (Fig. 7*C*). Moreover, Nakano *et al.* (30) revealed that, using a large-scale transcriptome analysis covering 27 types of immune cells from SLE and healthy donors, the *Pax5* gene expression was decreased in nonswitched memory B cells of the SLE patients. Thus, H3K4me1 appears to be crucial for maintaining naive B cells through an enhanced *Pax5* gene expression (Fig. 8).

In summary, we applied an LC–ESI–MS/MS-based method for the comprehensive analysis of lysine and histidine modifications to evaluate protein PTMs associated with SLE. This approach allowed us to identify lysine monomethylation as a unique alteration in PTMs of histones in SLE mouse splenocytes. The results of an adductome analysis also led to the hypothesis that dysregulation of histone lysine monomethylation may play a significant role in the pathophysiology of SLE. In particular, the discovery of the reduction of the H3K4me1 associated with SLE suggests that epigenetic interventions might be one of the effective therapeutic methods against SLE and other autoimmune diseases. Furthermore, our work may also provide a systematic workflow to aid in the development of neoantigen-based diagnoses and treatments for SLE and other autoimmune diseases. Taken together, this study provides a general protocol for systematically screening PTM-related neoantigens in patients with autoimmune diseases and advances our understanding of SLE pathogenesis.

## Experimental procedures

### Materials

All reagents used in the study were of analytical grade and obtained from commercial sources.

### Mice

All the experiments were performed according to the guidelines of the Animal Usage Committee of the Faculty of Agriculture, The University of Tokyo, and approved by the committee (permission no.: P19-020 and P21-025). Female BALB/c mice, female MRL-Mpj (MRL/MpJJmsSlc-+/+) mice, and female MRL-lpr mice (MRL/MpJJmsSlc-*lpr/lpr*) were purchased from Japan SLC. The mice were housed in a temperature-controlled pathogen-free room with light from 8:00 to 20:00 (daytime) and had free access to standard food and water. For the quantitative analysis, the mice (n = 3) were sacrificed, and the lung, liver, heart, kidney, brain, and spleen were harvested, flash frozen, and stored at −80 °C until processing. Blood was immediately treated with EDTA, and the plasma and RBC were fractionated by centrifugation (1200*g*, 4 °C, 10 min). The RBC fraction was washed three times with equal volumes of PBS.

### Protein adductome analysis using LC–ESI–MS/MS

The mouse tissues were homogenized in radioimmunoprecipitation assay buffer containing 50 mM Tris–HCl (pH 7.4), 150 mM NaCl, 1% Triton X-100, 0.5% sodium deoxycholate, 0.1% SDS, 1 mM EDTA, and 10 mM sodium fluoride. The samples were sonicated, then centrifuged at 12,000*g* for 5 min, and the supernatants were used as the cellular protein solution. Protein samples (4 mg) were reduced with 100 mM NaBH_4_ at room temperature for 3 h and extracted by the addition of 1 vol chloroform–methanol (1:3, v/v). Tubes were agitated using a vortex mixer and centrifuged at 6100*g* for 10 min. An interphase was formed, and the top fluid above was removed. Methanol (400 μl) was added to the samples and then centrifuged at 6100*g* for 10 min. The clear organic fluid was removed, and the pellet was dried by a centrifugal evaporator. For acid hydrolysis, the samples were hydrolyzed with 6 N HCl for 24 h at 110 °C. The resulting solution was neutralized with NaOH. For enzymatic digestion, the samples were suspended in 20 mM Tris–HCl buffer (pH 7.4) (500 μl) and exhaustively hydrolyzed by pronase (Merck) and leucine aminopeptidase (Sigma). Pronase (50 μl, 2 mg/ml) was added, and the samples were incubated at 37 °C for 24 h. The pH was then adjusted to 8.5, and MgCl_2_ was added to a final concentration of 5 mM. An activated leucine aminopeptidase solution (55 μl) was subsequently added. Leucine aminopeptidase was activated by incubating 225 μg/ml in 10 mM Tris–HCl buffer (pH 8.5) with 1 mM MgCl_2_ for 2 h at 40 °C. After the hydrolysis, the samples were partially separated by Oasis MCX cartridges (Waters) according to previous research (31). Briefly, (i) Equilibration: the cartridge was conditioned with 1 ml of methanol followed by 1 ml of acidified water (1% formic acid [FA]). (ii) Loading: the acidified samples (0.2% FA) were loaded for MCX. (iii) Washing: 1 ml of methanol containing 10% water and 0.1% FA was used and repeated twice. (iv) Elution: 1 ml of methanol containing 6% NH_4_OH was used for elution and repeated twice. Subsequently, the eluted solutions were dried by a centrifugal evaporator and redissolved in 1 ml of 50% acetonitrile and subjected to an adductome analysis using a TQD triple-stage quadrupole mass spectrometer (Waters) equipped with an ACQUITY ultraperformance LC system (Waters). The sample injection volumes of 5 μl each were separated on a Scherzo SM-C18 column (150 × 3.0 mm) (Imtakt) at the flow rate of 0.3 ml/min. A sample was separated by solvent A (H_2_O containing 0.1% FA) and solvent B (acetonitrile containing 0.1% FA) with a gradient elution of 5 to 100% solvent B in 8 min, then washed the column with 100% solvent B for 3 min. SRM was performed in the positive ion mode using nitrogen as the nebulizing gas. The experimental conditions were as follows: ion source temperature, 120 °C; desolvation temperature, 350 °C; cone voltage, 25 V; collision energy, 25 eV; desolvation gas flow rate, 700 l/h; cone gas flow rate, 50 l/h; and collision gas, argon. The strategy was designed to detect the product ion (*m/z* 84.0, for lysine adducts; *m/z* 110.0, for histidine adducts) from positively ionized adducts by monitoring the samples transmitting their [M + H]^+^ >84.0 (for lysine adducts) and [M + H]^+^ >110.0 (for histidine adducts) transitions. Twenty-five SRMs were analyzed simultaneously, shifting the *m/z* by 1.0 in each SRM. In total, nine cycles of analysis were performed to detect precursor ions in the *m/z* 126 to 350 range.

### Synthesis of mmeK

To a solution of l-lysine hydrochloride (20 mg) in 500 μl of water, 1 ml of benzoic anhydride (25 mg) in dimethylformamide was added. The solution was stirred overnight at room temperature, and *N*^*α*^-benzoyl-L-lysine was purified by reverse-phase HPLC. To a solution of *N*^*α*^-benzoyl-mmeK (10 mM) in 1 ml of water, 1.5 μl of formaldehyde (18%) was added. The solution was stirred for 1 h at room temperature, and sodium cyanoborohydride (3.1 mg) was added. The solution was stirred for 1 h at room temperature, and *N*^*α*^-benzoyl-mmeK was purified by reverse-phase HPLC. The product was hydrolyzed with 6 N HCl for 24 h at 110 °C. The resulting solution was neutralized with NaOH. After the neutralization, mmeK was purified by reverse-phase HPLC. [^13^C_6_, ^15^N_2_] mmeK was synthesized by the same methods. Purification of the lysine derivatives was performed by reverse-phase HPLC equipped with a Sunniest RP-AQUA column (5 μm, 10 × 250 mm; ChromaNik Technologies). The lysine derivatives were eluted with a gradient of water containing 0.1% (v/v) TFA (solvent A) and acetonitrile containing 0.1% TFA (solvent B) at the flow rate of 4 ml/min (0–5 min, 5% B and 5–30 min, 5–95% B). The product corresponding to mmeK was characterized by ^1^H-NMR and ^13^C-NMR. NMR spectra were recorded at 21 °C by a JNM-ECA500 II (JEOL). ^1^H-NMR (500 MHz, D_2_O): δH 1.57 to 1.70 (6H, m), 1.81 to 1.95 (4H, m), 2.57 (3H, s), 2.90 (2H, t), 3.37 (1H, t); ^13^C-NMR (125 MHz, D_2_O): δC 22.32, 26.25, 30.05, 39.25, 53.34, 48.47, 174.43.

### Quantification of mmeK by LC–ESI–MS/MS

The protein samples were extracted by the addition of 1 vol chloroform–methanol (1:3, v/v). The tubes were agitated by a vortex mixer and centrifuged at 6100*g* for 10 min. An interphase was formed, and the top fluid was removed. Methanol (400 μl) was added to the samples and then centrifuged at 6100*g* for 10 min. The clear organic fluid was removed, and the pellet was dried by a centrifugal evaporator. The samples were hydrolyzed with 300 μl of 6 N HCl for 24 h at 110 °C. The internal standard, [^13^C_6_, ^15^N_2_] mmeK, was added to the samples prior to the acidic hydrolysis. The resulting solution was neutralized with NaOH. After the neutralization, the samples were purified by an Oasis MCX. The eluted samples were then dried and dissolved in 50% acetonitrile and analyzed by a TQD triple-stage quadrupole mass spectrometer (Waters) equipped with an ACQUITY ultraperformance LC system (Waters). The sample injection volumes of 5 μl each were separated on a Scherzo SM-C18 column (150 × 3.0 mm) (Imtakt) at the flow rate of 0.3 ml/min. A discontinuous gradient was used by solvent A (H_2_O containing 0.1% FA) with solvent B (acetonitrile containing 0.1% FA) as follows: 5% B at 0 min, 100% B at 8 min, and 100% B at 13 min. SRM was performed in the positive ion mode using nitrogen as the nebulizing gas. The experimental conditions were as follows: ion source temperature, 120 °C; desolvation temperature, 350 °C; cone voltage, 10 V; collision energy, 10 eV; desolvation gas flow rate, 700 l/h; cone gas flow rate, 50 l/h; and collision gas, argon. The monitored SRM transitions were as follows: [^13^C_6_, ^15^N_2_] mmeK, *m/z* 169 > 90; mmeK, *m/z* 161 > 84. The amounts of mmeK were quantified by the ratio of the peak area of the target adducts and of the stable isotope.

### Western blotting

The samples were separated by 10% SDS-PAGE and transferred to a polyvinylidene difluoride membrane (Millipore). The membrane was incubated for 1 h with Blocking One (Nacalai Tesque) to block any nonspecific binding. The membrane was then incubated overnight at 4 °C with antibodies against mmeK (14679; Cell Signaling), histone H2B (5HH2-2A8; Millipore), histone H3 (D1A2; Cell Signaling), H3K4me1 (D1A9; Cell Signaling), H3K4me2 (C64G9; Cell Signaling), H3K4me3 (C42D8; Cell Signaling), H3K9me1 (D1P5R; Cell Signaling), H3K27me1 (28H59L67; Invitrogen), H3K36me1 (D9J1D; Cell Signaling), and H3K79me1 (A-4043-025; EPIGENTEK) in 10% Blocking One-TBST, followed by an incubation for 1 h at room temperature with horseradish peroxidase–conjugated secondary antibodies (1:2000 dilution, anti-rabbit IgG; Cell Signaling, #7074). Detection was performed using the SuperSignal West Pico Chemiluminescent Substrate (Thermo Fisher Scientific) and LAS4000 (Cytiva). The data were analyzed and quantified by ImageJ Fiji software, version 1.52n.

### Size-exclusion chromatography

The samples were centrifuged at 12,000*g* for 5 min. The supernatants were further purified by size-exclusion chromatography using an AKTA pure 25 (Cytiva) equipped with a Superose Increase 6 10/300 GL column (Cytiva) equilibrated with 50 mM phosphate buffer (pH 7.0) with 300 mM NaCl.

### Proteomic analysis by MS

Protein bands were excised from the SDS gel and subjected to in-gel digestion followed by a method described previously (32). The digested peptides were analyzed by nano-flow reverse-phase LC followed by tandem MS using a Q-Exactive hybrid mass spectrometer (Thermo Fisher Scientific). The capillary reverse-phase HPLC–MS/MS system comprised a Dionex U3000 gradient pump equipped with a VICI CHEMINERT valve. The Q-Exactive mass spectrometer was equipped with a nanoelectrospray ionization source (AMR). The desalted peptides were loaded into a separation capillary C18 reverse-phase column (NTCC-360/100-3-125, 125 × 0.1 mm; Nikkyo Technos). Peptide spectra over a mass range of *m/z* 350 to 1800 were recorded using an Xcalibur 3.0.63 system (Thermo Fisher Scientific). MS spectra were subsequently recorded, followed by 10 data-dependent high-energy collisional dissociation MS/MS spectra generated from the 10 highest intensity precursor ions.

### Data analysis

Mus_musculus_canonical_uniprot-download_2022.09.24.fasta and cRAP for contaminants (http://www.thegpm.org/crap/) were used. MS/MS spectra were interpreted, and peak lists were generated using Proteome Discoverer 2.2.0.400 (Thermo Fisher Scientific). Searches were performed using SEQUEST (Thermo Fisher Scientific). Search parameters were set as follows: trypsin was selected as the proteolytic enzyme with a maximum of two missed cleavages, a precursor mass tolerance of 10 ppm, and an MS/MS tolerance of 0.02 Da; carbamidomethylation of Cys was set as a fixed modification, and Met oxidation was set as a variable modification. Peptide identifications were accepted based on significant Xcorr values (high-confidence filter).

### Isolation of splenocytes

To obtain a single splenocyte suspension, mouse spleens were syringe homogenized and the cells were passed through a 100 μm cell strainer using RPMI1640+. All subsequent steps were conducted at 4 °C or on ice. The collected cells were pelleted in tubes by centrifugation at 300*g* for 5 min. The RBCs were lysed using 1× RBC lysis buffer (Invitrogen; 00-4333-57) for 1 min. The splenocytes were subsequently centrifuged at 300*g* for 5 min, resuspended in the medium, and counted.

### *In vitro* cell culture

The splenocytes were isolated from MRL-Mpj or MRL-lpr mice, seeded at 2 × 10^5^ cells/well in a 48-well plate in 500 μl of RPMI1640+ with SP2509 (Adooq BioScience LLC; ADQ-A14443-5-5), and incubated for 48 h at 37 °C. After 48 h, all the cells were harvested, and the supernatant was collected for quantification of the secreted immunoglobulin. Immune cell subsets from BALB/c mice were isolated using immunomagnetic beads. Briefly, splenocytes (1 × 10^7^ cells) suspended in the MACS buffer (PBS/2 mM EDTA/0.5% bovine serum albumin) were incubated with 10 μl of CD4 (L3T4) microbeads (Miltenyi Biotec), CD8 (Ly-2) microbeads (Miltenyi Biotec), or CD45 (B220) microbeads (Miltenyi Biotec) for 15 min at 4 °C. The cells were washed by adding 0.5 ml of the MACS buffer and centrifuged at 300*g* for 10 min. After aspirating the supernatant, the microbead-bound cells were resuspended in 0.5 ml of the buffer, and the cells were enriched using LS MACS columns (Miltenyi Biotech) according to the manufacturer's instructions. The purified B cells were subsequently cultured in RPMI1640 with the addition of IL-4 (PeproTech; 214-14-20, 30 ng/ml), R848 (a TLR7 agonist; MedChemExpress; HY-13740, 100 ng/ml), SP2509, and EG1 (Selleck; S0858, 50 μM). Cultures were established in 48-well plates with 2 × 10^5^ cells/well in 500 μl of medium at 5% CO_2_ and 37 °C. After 48 h, all the cells were harvested, and the supernatant was collected for quantification of the secreted immunoglobulin.

### Flow cytometry

The cells were washed and resuspended in fluorescence-activated cell sorting (FACS) buffer (PBS containing 2% fetal bovine serum). Single-cell suspensions in the FACS buffer were Fc-blocked using anti-mouse CD16/32 (TruStain fcXTM; clone: 93) at 4 °C for 15 min. Surface staining was performed using Abs including anti-mouse CD5-FITC (BioLegend; clone: 53-7.3), anti-mouse CD19-PECy7 (BioLegend; clone: 6D5), and anti-mouse CD138-PE (BioLegend; clone: 281-2) at 4 °C for 15 min. Dead cells were identified by the scatter properties. Data were acquired by FACS Verse (BD Bioscience) and subsequently analyzed using FlowJo software, version 10 (BD Bioscience).

### Quantification of immunoglobulin

The total immunoglobulin levels were measured by sandwich ELISA. The goat anti-mouse IgG (Bethyl; A90-131A) or anti-mouse IgM (SouthernBiotech; 1020-01) was used as a capture antibody. A 100 μl aliquot of capture antibody (1:500 dilution) was added to each well of a 96-well microtiter plate and incubated overnight at 4 °C. The plate was then washed with PBS with Tween-20 (PBST) and blocked using 20% Blocking One (Nacalai Tesque; 03953-95) for 1 h at room temperature. After discarding the supernatants and washing with PBST, a 100 μl aliquot of either the diluted mice sera, diluted monoclonal antibodies, or diluted culture media was added to each well and incubated for 2 h at room temperature. After discarding the supernatants and washing with PBST, 100 μl of a 1:5000 dilution of goat anti-mouse IgG conjugated to horseradish peroxidase (abcam; ab97265) or anti-mouse IgM conjugated to horseradish peroxidase (SouthernBiotech; 1021-05) in PBST was added. After incubation for 1 h at 37 °C, the supernatant was discarded followed by washing, and the enzyme-linked Ab bound to the well was developed by 1-Step Ultra TMB-ELISA. The reaction was terminated by the addition of 2 N sulfuric acid (50 μl/well), and the absorbance at 450 nm was read using a micro-ELISA plate reader. The IgG mouse ELISA standard (Invitrogen; 39-50400-65) and IgM mouse ELISA standard (Invitrogen; 39-50470-65) were used as the immunoglobulin standard. Subsequently, twofold serial dilutions of the standard were performed with PBS to make the standard curve (250 ng/ml, 125 ng/ml, 62.5 ng/ml, 31.25 ng/ml, 15.625 ng/ml, 7.8125 ng/ml, 3.90625 ng/ml, and blank as 0 ng/ml).

### Quantitative PCR

The total RNA was extracted from the cells using the FastGene RNA Basic Kit (NIPPON Genetics) and reverse transcribed using ReverTra Ace (Toyobo) according to the supplier’s instructions. The PCR amplification was performed using the THUNDERBIRD SYBR qPCR Mix (Toyobo) and LightCycler 96 system (Roche). The expression levels were normalized using β-actin as an endogenous control gene. The applied primer sequences are shown in Table S3.

### Chromatin immunoprecipitation

Isolated splenic B cells were lysed in 1 ml of lysis buffer (50 mM Hepes [pH  7.9], 140 mM NaCl, 1 mM EDTA, 10% glycerol, 0.5% NP-40, 0.25% Triton X-100, and 1× protease inhibitors) for 10 min on ice. The lysed cells were centrifuged at 4000 rpm for 5 min at 4 °C and washed twice with 500 μl of cold wash buffer (10 mM Tris–HCl [pH  7.5], 200 mM NaCl, 1 mM EDTA [pH  8.0], 1×protease inhibitors). The pellet was resuspended in 500 μl of cutting buffer (10 mM Tris–HCl [pH 7.5], 15 mM NaCl, 60 mM KCl, and 2 mM CaCl_2_). MNase (Takara) was added at 1 U per 10^6^ cells, and the nuclei were digested at 37 °C for 90 min. The MNase reaction was stopped by adding EDTA to a final concentration of 20 mM. The digested nuclei were centrifuged at 1300*g* for 5 min at 4 °C. The pellet was resuspended with 500 μl of TE buffer and incubated for 1 h with mixing by a rotator. The samples were centrifuged at 13,000*g* for 5 min at 4 °C, and the supernatants were collected. For input, 1% of the material was set aside. The samples were diluted by ChIP buffer (10 mM Tris–HCl [pH 7.5], 150 mM NaCl, 5 mM EDTA, and 0.05% Tween-20), supplemented with protein G-sepharose, and incubated for 1 h at room temperature with mixing by a rotator. The samples were centrifuged at 12,000*g* for 5 min at 4 °C. The cleared extracts were supplemented with 10 μl of normal IgG (2729; Cell Signaling) or Anti-H3K4me1 (D1A9; Cell Signaling) and protein G-sepharose. The samples were incubated overnight at 4 °C with mixing by a rotator. The beads were washed five times with ChIP buffer. The enriched DNA was eluted in 70 μl of elution buffer (1% SDS, 100 mM NaHCO_3_) supplemented with 4.8 μl of 5 M NaCl and 40 μg of proteinase K (Sigma) overnight at 50 °C. The DNA was isolated using the PureLink PCR purification kit (Invitrogen).

### Statistics and reproducibility

All the results were confirmed by three independent experiments, with the data of one representative experiment being presented. The data are represented as the mean with the SD of triplicate samples as described in the respective figure legends. Statistical analyses were performed using Prism Software, v6.07 (GraphPad), with a *p* value of <0.05 being considered significant. Differences between the two groups were analyzed by a two-tailed Student’s *t* test. Multiple comparisons between groups were made using Tukey–Kramer tests or Dunnett’s test. Statistical significance between two groups was determined using a multiple *t* test using the Benjamini, Krieger, and Yekutieli method to correct for multiple comparisons, using a desired false discovery rate of 1%.

## Data availability

All data presented are contained within the main article and supporting information.

## Supporting information

This article contains supporting information.

## Conflict of interest

The authors declare that they have no conflicts of interest with the contents of this article.
