# Supplementary material for: Adductome-based identification of lysine monomethylation as a key post-translational protein modification in autoimmune diseases
Source: J Biol Chem. 2025 Sep 4;301(10):110684. doi: 10.1016/j.jbc.2025.110684 (PMC12509770; doi:10.1016/j.jbc.2025.110684)
Supplement: Supporting information [file mmc2.pdf]

Supporting Information

Adductome-based identification of lysine mono-methylation as a key post-translational protein modification in autoimmune diseases

Kosuke Yamaguchi[1], You-Yun Hu[1], Kaito Kawajiri[1], Masanori Itakura[1], Fumie Nakashima[2], Takahiro Shibata[2], and Koji Uchida[1]

[1] Graduate School of Agricultural and Life Sciences, The University of Tokyo, Tokyo, Japan
[2] Graduate School of Bioagricultural Sciences, Nagoya University, Nagoya, Japan

Supplementary Figures

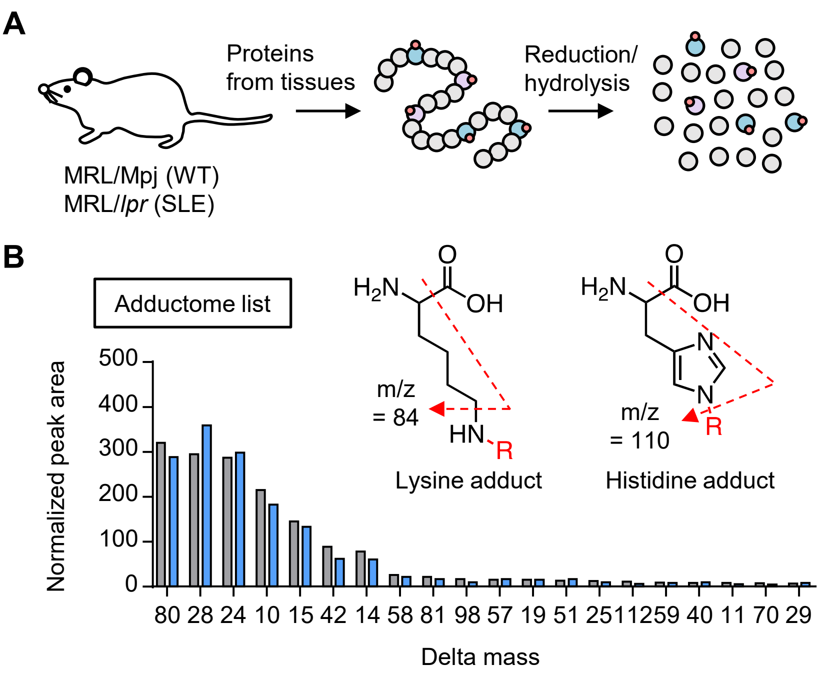

Fig. S1 Strategy of lysine and histidine adductome analysis.

A, In this study, proteins from tissues were reduced with sodium borohydride ($NaBH_4$) to stabilize the unstable adducts and then hydrolyzed to free (modified) amino acids by enzymatic digestion. The resulting free amino acid mixture was subjected to LC-ESI-MS/MS analyses. **B,** The strategy was designed to detect the product ion of *m/z* 84 (loss of NH_3 from the lysine immonium ion) from positively ionized lysine adducts and *m/z* 110 (immonium ion of histidine) from the positively ionized histidine adducts by LC-ESI-MS/MS. The MS data could be visualized as an adductome list.

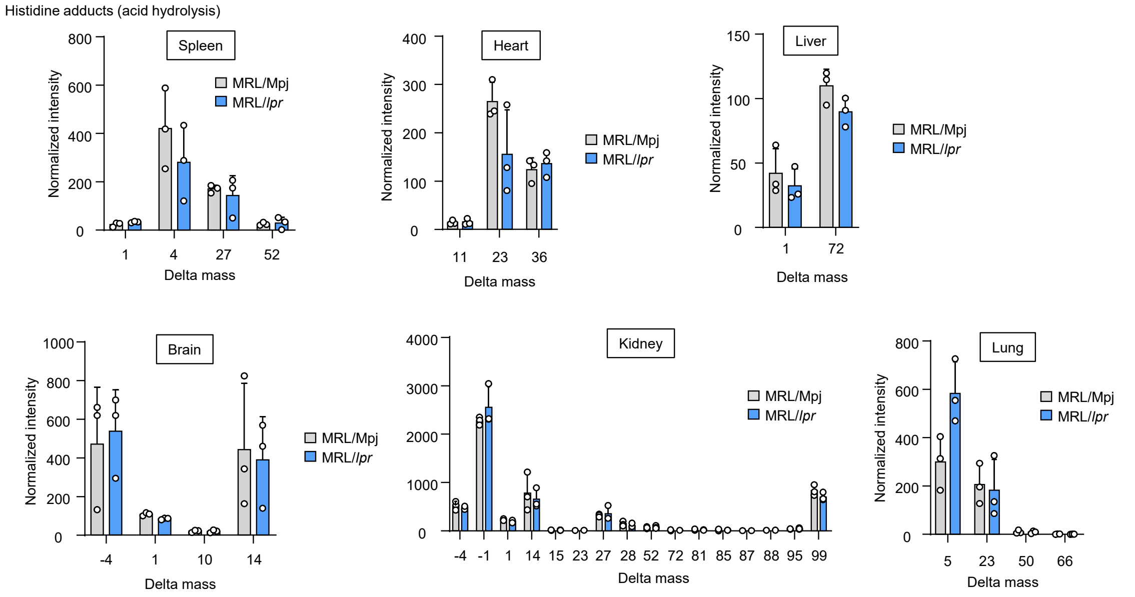

Fig. S2 Adductome analysis of histidine adducts from acid-hydrolyzed samples. The delta mass clusters in each of the tissue samples (n = 3). Among the delta mass shifts of -30 to 194, only those that could be detected are shown. Statistical significance between each group was determined using a multiple *t*-test.

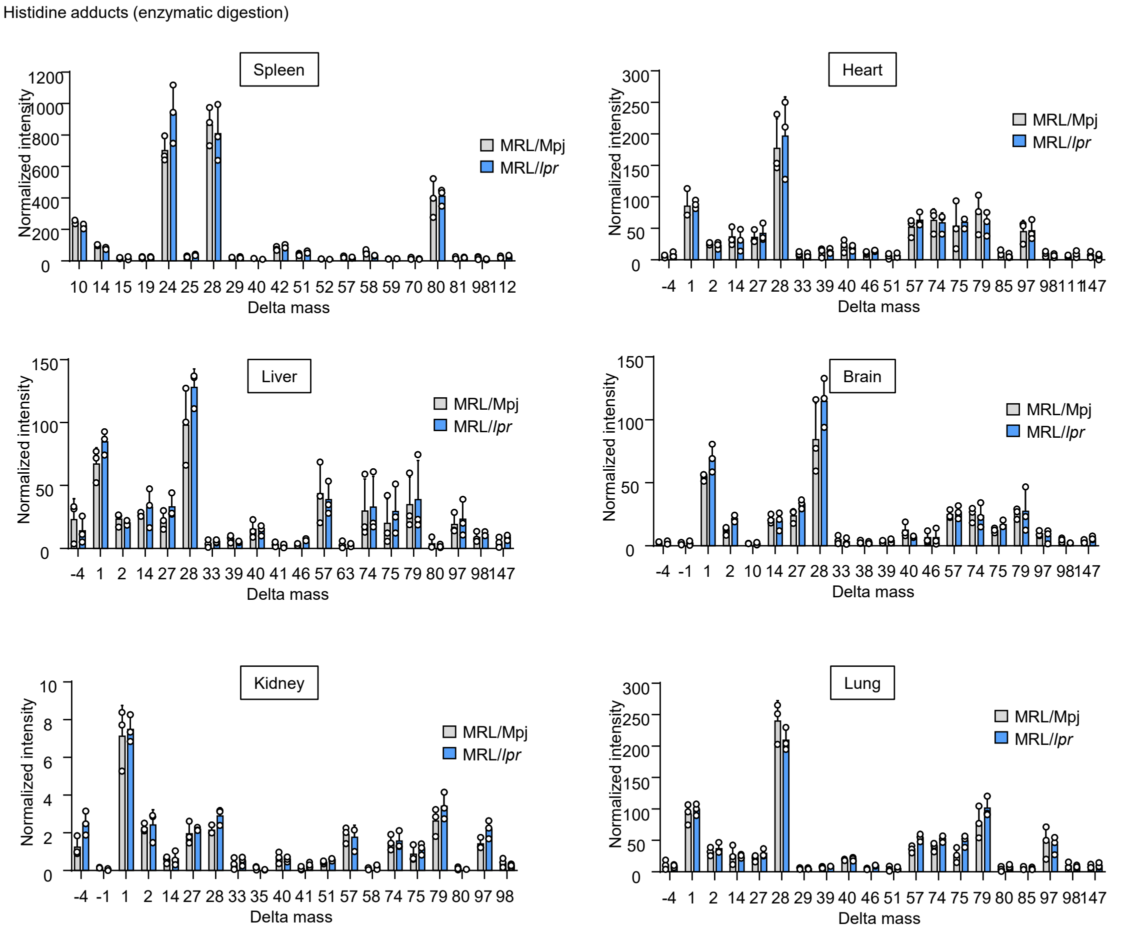

Fig. S3 Adductome analysis of histidine adducts from enzymatically digested samples.

The delta mass clusters with the highest frequencies in each of the tissue samples (n = 3). Among the delta mass shifts of -30 to 194, top 20 are shown. Statistical significance between each group was determined using a multiple *t*-test.

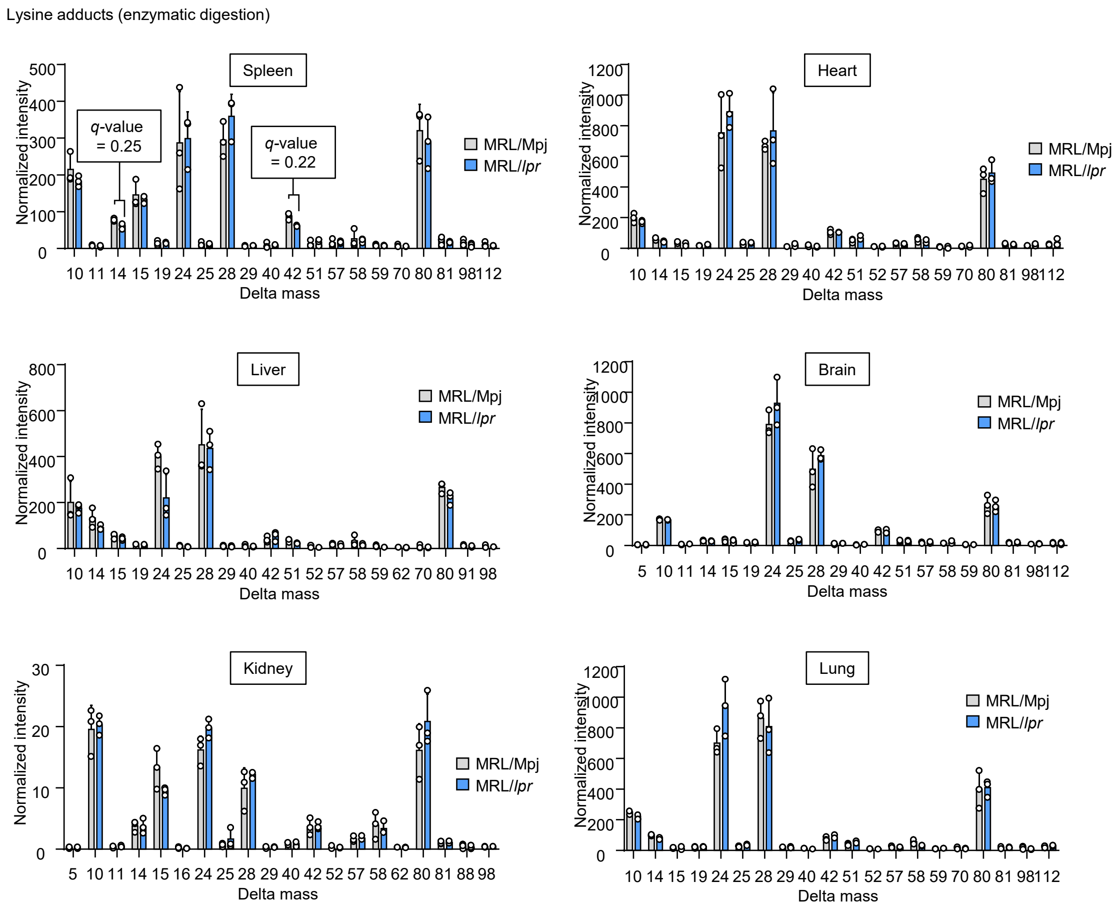

Fig. S4 Adductome analysis of lysine adducts from enzymatically digested samples. The delta mass clusters with the highest frequencies in each of the tissue samples (n = 3). Among the delta mass shifts of -21 to 203, top 20 are shown. Statistical significance between each group was determined using a multiple *t*-test.

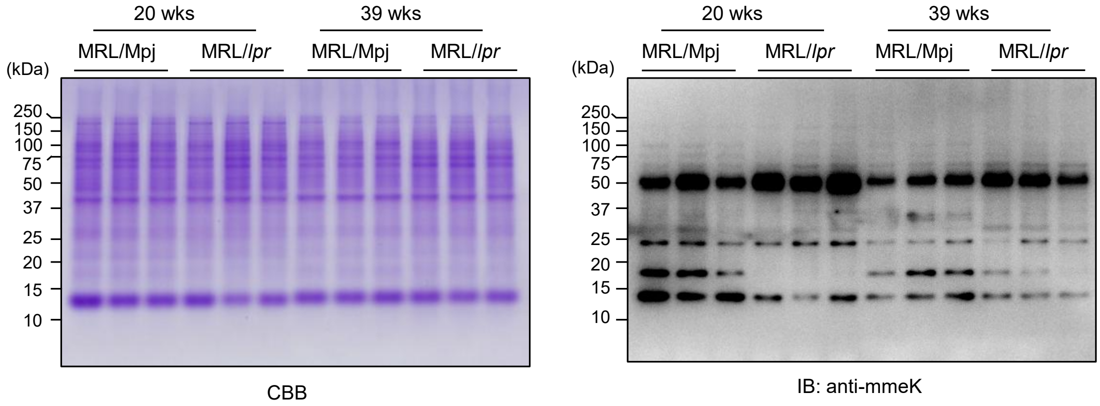

Fig. S5 Age-independent reduction of mmeK-containing proteins in SLE-prone MRL-lpr mice

Immunoblotting showing mmeK-containing protein levels in the spleen of MRL-Mpj and MRL-lpr mice. *Left panel*, SDS-PAGE and Coomassie Brilliant Blue (CBB) staining; *right panel*, immunoblot analysis with anti-mmeK antibody.

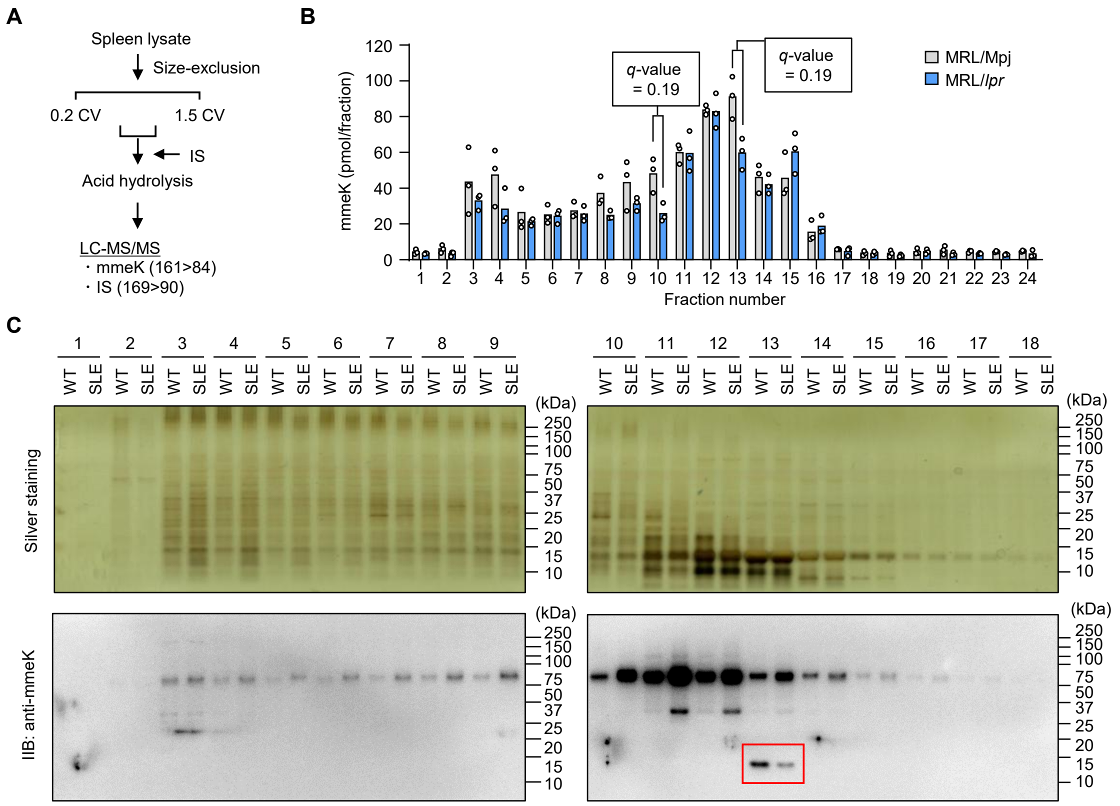

Fig. S6 Analysis of mmeK-containing proteins using size-exclusion chromatography. **A,** Schematic of biochemical strategy to identify mmeK-containing proteins. **B,** Quantitative identification of mmeK in the splenic lysates separated by size-exclusion chromatography. The mmeK was quantified using LC-ESI-MS/MS coupled with a stable isotope dilution method. The data are shown as the mean ± SD (n = 3, biologically independent experiments). Statistical significance between each group was determined using a multiple *t*-test. **C,** Immunoblotting showing mmeK-containing protein levels in the splenic lysates separated by size-exclusion chromatography. *Upper panel*, SDS-PAGE and silver staining; *lower panel*, immunoblot analysis with anti-mmeK antibody.

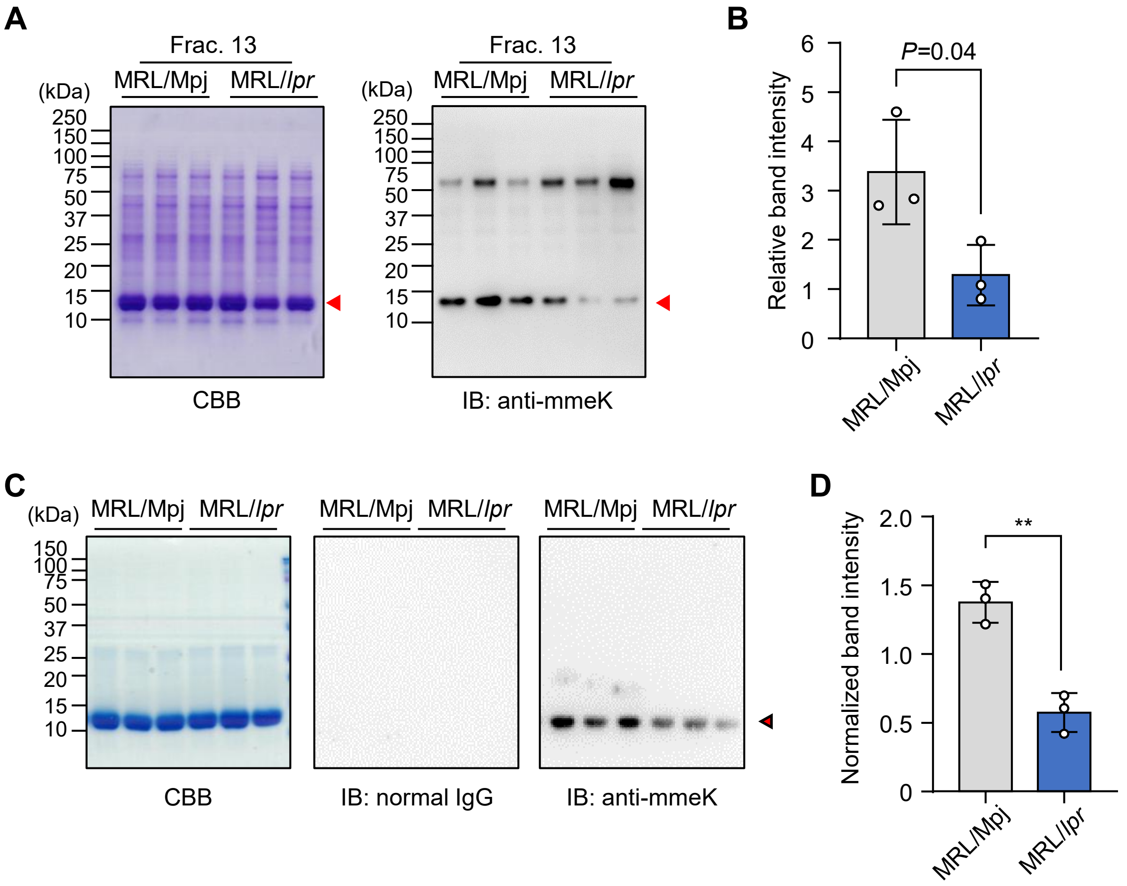

Fig. S7 Analysis of mmeK-containing proteins contained in fraction No. 13.

A, Immunoblotting showing mmeK-containing protein levels in fraction No. 13. *Left panel*, SDS-PAGE and Coomassie Brilliant Blue (CBB) staining; *right panel*, immunoblot analysis with anti-mmeK antibody. Arrowhead, candidate of band B. **B,** Quantification of band B shown in panel **A**. The band B intensity (immunoblotting) was measured and normalized to the total band B (CBB staining). The data are shown as the mean ± SD (n = 3, biologically independent experiments). Student's *t* test (two-sided). **C,** Immunoblotting showing mmeK-containing protein levels in the RBC fraction. *Left panel*, SDS-PAGE and Coomassie Brilliant Blue (CBB) staining; *middle panel*, immunoblot analysis with normal IgG; *right panel*, immunoblot analysis with anti-mmeK antibody. Arrowhead, candidate of band B. **D,** Quantification of band B shown in panel **C**. The band B intensity (immunoblotting) was measured and normalized to the total band B (CBB staining). The data are shown as the mean ± SD (n = 3, biologically independent

experiments). Student's *t* test (two-sided).

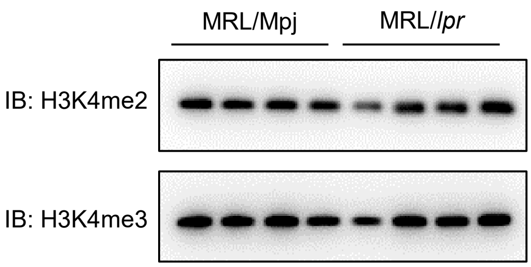

Fig. S8 Analysis of H3K4 methylation state.

Immunoblotting showing H3K4 methylation in B cells. *Upper panel*, immunoblot analysis with anti-H3K4me2 antibody; *lower panel*, immunoblot analysis with anti-H3K4me3 antibody.

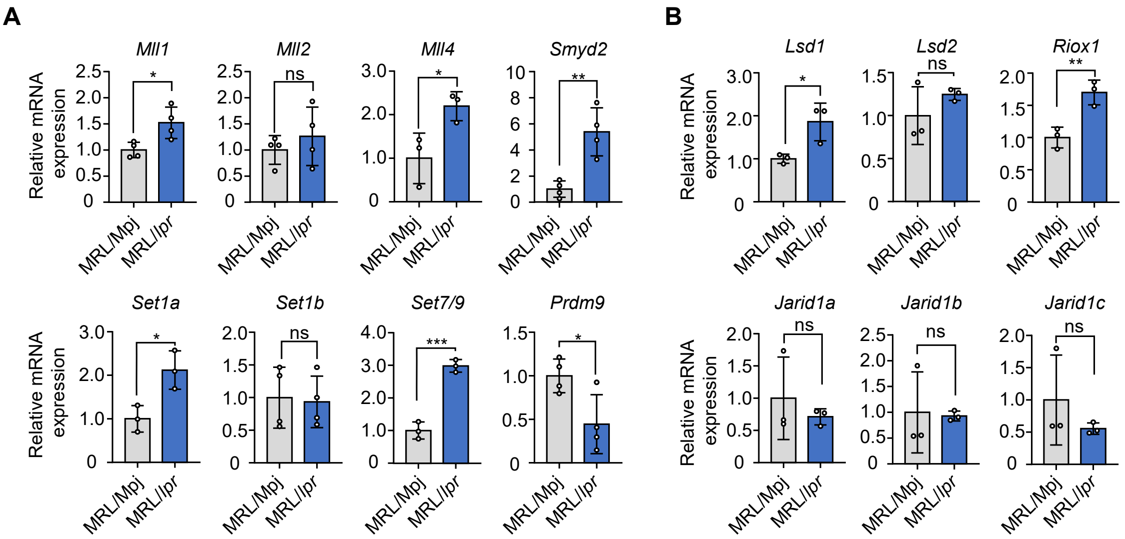

Fig. S9 mRNA expression levels of H3K4 methylation-related enzymes.

A, Quantification of H3K4 methyltransferase transcripts. The mRNA expression levels of the H3K4 methyltransferases were measured and normalized to *Actb*. The data are shown as the mean ± SD (n = 3 or 4, biologically independent experiments). Student's *t* test (two-sided). $*p < 0.05$; $**p < 0.01$; $***p < 0.001$. **B,** Quantification of H3K4 demethylase transcripts. The mRNA expression levels of the H3K4 demethylases were measured and normalized to *Actb*. The data are shown as the mean ± SD (n = 3 or 4, biologically independent experiments). Student's *t* test (two-sided). $*p < 0.05$; $**p < 0.01$.

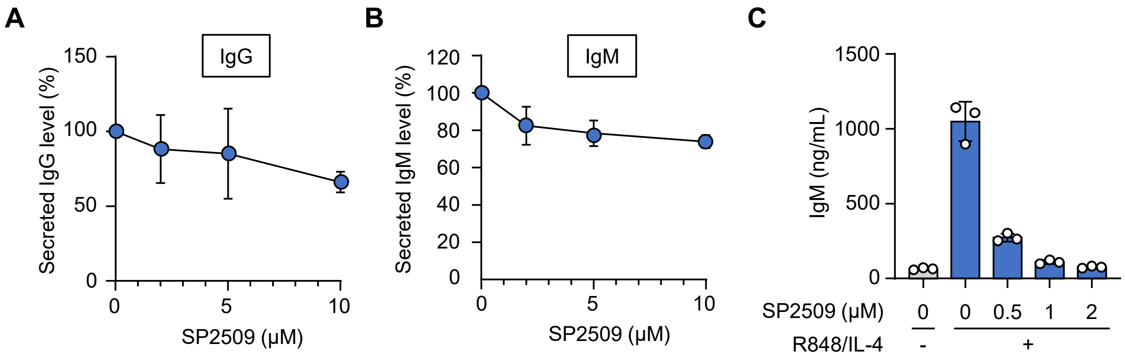

Fig. S10 The effects of LSD1 inhibitor SP2509 on B cell differentiation.

A, Quantification of secreted IgG in MRL-lpr mice-derived splenocytes cultures. The amount of IgG without SP2509 was regarded as 100%. The data are shown as the mean ± SD (n = 3, biologically independent experiments). **B,** Quantification of secreted IgM in MRL-lpr mice-derived splenocytes cultures. The amount of IgM without SP2509 was regarded as 100%. The data are shown as the mean ± SD (n = 3, biologically independent experiments). **C,** Quantification of secreted IgM in B cell cultures. The data are shown as the mean ± SD (n = 3, biologically independent experiments).

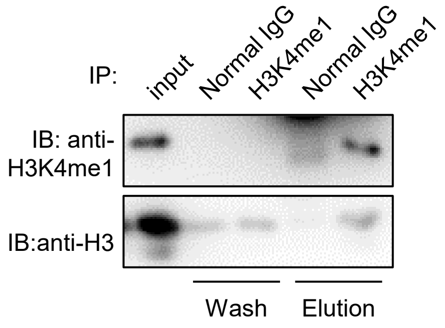

Fig. S11 Immunoprecipitation assay using anti-H3K4me1 antibody.

Immunoblotting showing H3 and H3K4me1 in the immunoprecipitated samples. *Upper panel*, immunoblot analysis with anti-H3K4me1 antibody; *lower panel*, immunoblot analysis with anti-H3 antibody.

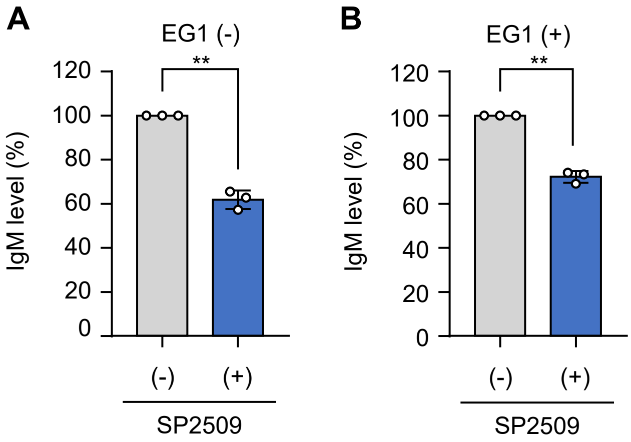

Fig. S12 The effects of PAX inhibitor EG1 on IgM secretion.

A, Quantification of secreted IgM in control B cell cultures. The B cells from BALB/c mice were stimulated with R848/IL-4 and cultured with SP2509 (0.5 μM) for 48 h. The amount of IgM without SP2509 was regarded as 100%. The data are shown as the mean ± SD (n = 3, biologically independent experiments). Student's *t* test (two-sided). $^{**}p < 0.01$. **B,** Quantification of secreted IgM in EG1-treated B cell cultures. The B cells from BALB/c mice were stimulated with R848/IL-4 and cultured with SP2509 (0.5 μM) and EG1 (30 μM) for 48 h. The amount of IgM without SP2509 was regarded as 100%. The data are shown as the mean ± SD (n = 3, biologically independent experiments). Student's *t* test (two-sided). $^{**}p < 0.01$.
